# Intelligence

**Published:** 1832-11

**Authors:** 


### INTELLIGENCE.

Westminster Medical Society. The members held their first meeting of this session on Saturday, the 20th of October, in the Hunterian Museum, Great Windmill street; when Dr. Copland and Mr. Pettigrew were elected joint presidents with Drs. Sigmond and Jewell.

---

Medico-Botanical Society. The first public meeting of the ensuing session will be held on Tuesday, the 13th of November, at eight P.M., in the Society's rooms, Sackville street.

---

Phrenology, London Hospital. The introductory lecture to the Phrenological Course will be given on Saturday, November 10, 1832, at eleven o'clock, by Mr. H. Haley Holm. A lecture will be delivered on the same day and hour each week, until the course, consisting of about twenty lectures, be completed.

---

In our announcement of Mr. Taylor's lectures on "Medical Jurisprudence," to be delivered in the Medical Theatre of Guy's Hospital, we should have said that the introductory lecture will be delivered on Tuesday, the 6th of November, at half-past three P.M. Most of the medical courses beginning in October occasioned the mistake.

---

REGULATIONS OF THE ROYAL COLLEGE OF SURGEONS, LONDON.

The Council of the College of Surgeons in Lincoln's Inn Fields require candidates for their diploma to bring proof

1. Of being twenty-two years of age.

2. Of having been engaged six years in the acquirement of professional knowledge.

3. Of having studied anatomy and physiology, by attendance on lectures and demonstrations, and by dissection, during two anatomical seasons.

*** An anatomical season is understood to extend from October to April inclusive, and to comprise at least 140 lectures on anatomy and physiology, occupying not less that one hour each, given on separate days; and at least one hundred demonstrations of the like duration, given in a similar manner; exclusive of dissections, of which distinct certificates are required.

4. Of having attended at least two courses of lectures on surgery, delivered in two distinct periods or seasons; each course to comprise not less than sixty lectures.

5. Of having attended lectures on the practice of physic, on chemistry, and on midwifery, during six months; and on botany and materia medica during three months.

6. Of having attended during twelve months the surgical practice of a recognised hospital in London, Dublin, Edinburgh, Glasgow, or Aberdeen; or for six months in any one of such hospitals, and twelve months in any recognised provincial hospital.

---

### LITERARY INTELLIGENCE.

In a few days will be published a new edition of Dr. Hooper's "Physician's Vade Mecum, or Manual of the Principles and Practice of Physic," very considerably enlarged, and brought down to the present state of medical science.

---

Regulations *to be observed by Students intending to qualify themselves to practise as Apothecaries in England and Wales.* (*Issued by the Apothecaries' Company, Sept.* 1832.)

Every candidate for a certificate to practise as an apothecary, will be required to produce testimonials.

Of having served an apprenticeship* of not less than five years to an apothecary;

Of having attained the full age† of twenty-one years;

And of good moral conduct.‡

* The apprenticeship must have been served with a person legally qualified to practise as an apothecary, either by having been in practice prior to or on the 1st of August, 1815, or by having received a certificate of his qualification from the Court of Examiners.

† As evidence of age, a copy of the baptismal register will be required in every case where it can possibly be procured.

‡ A testimonial of moral character from the gentleman to whom the candidate has been an apprentice, will always be more satisfactory than from any other person.

Students whose attendance on lectures commenced on or after January 1831, must, in addition to these testimonials, adduce proof of having devoted at least two years to an attendance on lectures and hospital practice; and of having attended the following courses of lectures:*

Chemistry, two courses; each course consisting of not less than forty-five lectures.

Materia Medica† and Therapeutics, two courses; each course consisting of not less than forty-five lectures.

Anatomy and Physiology, two courses; Anatomical Demonstrations, two courses; of the same extent as required by the Royal College of Surgeons of London.

Principles‡ and Practice of Medicine, two courses; each course consisting of not less than forty five lectures, to be attended subsequently to the termination of the first course of lectures on chemistry, materia medica, and anatomy and physiology.

Botany,§ one course; consisting of not less than thirty lectures, to be attended between the 1st of April and 31st of October.

Midwifery, and the Diseases of Women and Children, two courses.

Forensic Medicine, one course, to be attended during the second year.

Students are likewise earnestly recommended to avail themselves of instruction in morbid anatomy.

The candidate must also have attended for twelve months, at least, the physician's practice at an hospital containing not less than sixty beds, and where a course of clinical lectures is given; or for fifteen months at an hospital wherein clinical lectures are not given; or for fifteen months at a dispensary connected with some medical school recognized by the court.‖ The whole of such attendance to be subsequent to the first year of attendance on lectures.

Students whose attendance on lectures commenced prior to the 1st of February 1828, will be admitted to examination in conformity with the regulations published in September 1826, viz. after an attendance on

One course of lectures on Chemistry;

One course of lectures on Materia Medica:

Two courses of lectures on Anatomy and Physiology;

Two courses of lectures on the Theory and Practice of Medicine;

And six months' physician's practice at an hospital, or nine months at a dispensary.

Those who began to attend lectures subsequently to the 1st of February 1828, and previously to the 1st of October of the same year, in conformity with the regulations of September 1827, viz. after an attendance on

One course of lectures on Chemistry;

* The lectures required in each course respectively, must be given on separate days.

† Or on three courses of lectures given by the professor of materia medica at this Hall (the candidates having been apprentices of members of the Society), each course consisting of not less than thirty lectures.

‡ In these schools where the courses of lectures are of six months' duration, students may commence their attendance on the lectures on the principles and practice of medicine, after having attended for three months the lectures on chemistry, materia medica, and anatomy.

§ Certificates of attendance on the lectures and demonstrations given at the Society's garden, and also at the herborizing for two entire seasons, will be received as equivalent from such candidates as have been apprentices of members of the Society.

‖ The court are willing to recognize dispensaries upon receiving a satisfactory assurance from the physicians of those institutions, that adequate arrangements exist for affording instruction to students in practical medicine.

One course of lectures on Materia Medica and Botany;

Two courses of lectures on Anatomy and Physiology;

Two courses of lectures on the Theory and Practice of Medicine: the last having been attended subsequently to the lectures on Chemistry and Materia Medica, and to one course at least of Anatomy;

And six months, at least, physician's practice at an hospital, or nine months at a dispensary: such attendance having commenced subsequently to the termination of the first course of lectures on the Principles and Practice of Medicine.

Those whose attendance on lectures commenced in October 1828, must have complied with the regulations of September 1828; viz. by having attended

Two courses of lectures on Chemistry;

Two courses of lectures on Materia Medica and Botany;

Two courses of lectures on Anatomy and Physiology;

Two courses of Anatomical Demonstrations;

Two courses of lectures on the Theory and Practice of Medicine: these last having been attended subsequently to one course of lectures on Chemistry, Materia Medica, and Anatomy;

And six months, at least, the physician's practice at an hospital (containing not less than sixty beds), or nine months at a dispensary: such attendance to have commenced subsequently to the termination of the first course of lectures on the Principles and Practice of Medicine.

All students who began to attend lectures in January 1829, are required to have attended the physician's practice at an hospital for nine months, or at a dispensary for twelve months, and also to have attended

Two courses of lectures on Midwifery, and the Diseases of Women and Children.

The testimonials of attendance on lectures and hospital practice must be given on a printed form, with which students may be supplied, on application at the undermentioned places: In London, at the beadle's office, at this Hall. In Edinburgh, at Messrs. Mac Lachlan and Stewart's, booksellers. In Dublin, at Messrs. Hodges and Smith's, booksellers. In the provincial towns, where there are medical schools, from the gentlemen who keep the register of the school. No other form of testimonial will be received; and no attendance on lectures will qualify a candidate for examination, unless the lecturer is recognised by the court. The names of the lecturers recognised by the court may be seen on application to the several gentlemen acting as registrars in the provincial schools, and at the beadle's office at the Hall. The teachers in London, Dublin, Edinburgh, Glasgow, and Aberdeen, recognised by the constituted medical authorities in those places respectively, are recognised by the court; and certificates given by the medical professors in the continental universities are also recognised and received by the court.

*Registration.* A book is kept at the Hall of the Society for the registration, at stated times, of the names of students, and the lecturers, hospitals or dispensaries, they attend. All students in London are required to appear personally, and to register the several classes for which they have taken tickets; and those only will be considered to have complied with the regulations of the court whose names and classes in the register correspond with the testimonials of the teachers. The book will be open for the registration during the first twenty-one days of the months of October, February, and June, from nine o'clock until two.

The court also require students at the provincial medical schools to register their names in their own hand-writing, and the classes they attend, with the registrar of each respective school, within fourteen days from the commencement of each course of lectures, and those students only will be deemed to have complied with the regulations whose names are so registered.

The registrar of each respective school is requested, as soon as may be convenient after the termination of each scholastic year, to send to the secretary of the court a list of the names of the students registered with him during that year.

*Names of Gentlemen having the care of Registers.*

Bath, R. T. Gower, Esq., John Spender, Esq., lecturers on anatomy.
Birmingham, W. Sands Cox, Esq. ditto.
Bristol, Dr. Wallis, Henry Clark, Esq , ditto.
Hull, Edward Wallis, Esq., Robert Craven, Esq., ditto.
Leeds, Charles Turner Thackrah, Esq., ditto.
Liverpool, William Gill, Esq., ditto.
Manchester, Jos. Jordan, Esq., Thos. Turner, Esq., ditto.
Sheffield, Wilson Overend Esq., W. Jackson, Esq., ditto.

Each student at his first registration will receive the printed form on which he is to obtain the certificates of his teachers.

*Examination.* Every person offering himself for examination must give notice in writing to the clerk of the Society on or before the Monday previously to the day of examination, and must also at the same time deposit all the required testimonials at the office of the beadle, where attendance is given every day, except Sunday, from nine until two o'clock.

Candidates will be admitted to examination in the order in which their names stand on the notice paper; and those neglecting to attend agreeably to their notice will, upon a subsequent application, be placed at the bottom of the list.

The examination of the candidate for a certificate of qualification to practise as an apothecary will be as follows:

1. In translating parts of Celsus de Medicinâ, or Gregory's Conspectus Medicinæ Theoreticæ, physicians' prescriptions, and the Pharmacopœia Londinensis; 2, in chemistry; 3, in materia medica and therapeutics; 4, in botany; 5, in anatomy and physiology; 6, in the principles and practice of medicine.*

The examination of a candidate for a certificate of qualification to act as an assistant to an apothecary, in compounding and dispensing medicines, will be as follows: 1, in translating physician's prescriptions and parts of the Pharmacopœia Londinensis; 2, in pharmacy and materia medica.

By the 22d section of the Act of Parliament, no rejected candidate for a certificate to practise as an apothecary can be readmitted to be examined until the expiration of six months from his former examination; and no rejected candidate as an assistant until the expiration of three months.

The Court meet in the Hall every Thursday, where candidates are required to attend at a quarter before four o'clock.

---

### *Regulations of the Navy Medical Board relating to Assistant Surgeons in the Royal Navy.*

*Victualling Office.*

The Right Honourable the Lords Commissioners of the Admiralty having been pleased to direct "that no person be admitted to be a candidate for the situation of assistant-surgeon in the royal navy, who shall not produce a certificate from one of the Royal Colleges of Surgeons of London, Edinburgh, or Dublin, of his fitness for that office; nor for that of surgeon, unless he shall produce a diploma, or certificate, from one of the said royal colleges, founded on an examination to be passed subsequently to his appointment of assistant surgeon, as to the candidate's fitness for the situation of surgeon in the navy: and that in every case the candidate producing such certificate, or diploma, shall also undergo a further examination before the medical commissioners of the Victualling Board, touching his qualifications in all the necessary branches and points of medicine and surgery for each of the steps in the naval medical service." The

* This branch of examination embraces an inquiry into the diseases of pregnant and puerperal women; and also into the diseases of children.

commissioners for victualling his Majesty's navy, &c. do hereby signify, for the information of those persons to whom it may relate, that these regulations and directions will be strictly adhered to in future: and further, that, previously to the admission of assistant surgeons into the navy, it will be required that they should have received a classical education, and possess in particular a competent knowledge of Latin; also

That they should have served an apprenticeship, or have been employed in an apothecary's shop for not less than two years.

That their age should not be less than twenty years, nor more than twenty-six years; and that they should be unmarried.

That they should have attended an hospital in London, Edinburgh, Dublin, or Glasgow, for twelve months; and

That they should have attended lectures, &c. on the following subjects, for periods not less than hereunder stated; observing, however, that such lectures will not be admitted for more than two different branches of science, by one individual, viz.

| | |
|---|---|
| Anatomy | 18 months |
| Surgery | 18 |
| Theory of Medicine | 12 |
| Practice of ditto | 12 |
| Chemistry | 6 |
| Materia Medica | 6 |
| Midwifery | 6 |
| Actual dissections of the human body | 6 |

Although the above are the only qualifications which are absolutely required in candidates for the appointment of assistant surgeon, a preference will be given to those who, by possessing a knowledge of diseases of the eye, and of any branch of science connected with the profession, such as botany, medical jurisprudence, natural philosophy, &c., appear to be more peculiarly eligible for admission into the service.

It is also to be observed that, by the rules of the service, no assistant surgeon can be promoted to the rank of surgeon until he shall have been three years in the former capacity; and the Board have resolved that not any diploma or certificate of examination from either of the aforesaid royal colleges shall be admitted towards the qualification for surgeon, unless the diploma or certificate shall be obtained on an examination passed after a period of not less than three years from the date of the party's admission into the service.

By command of the Board,

M. Waller Clifton.

---

### OBITUARY.

### METEOROLOGICAL JOURNAL,

By Messrs. Harris and Co. Mathematical Instrument Makers, 50, High Holborn.

| | Moon. | Rain gauge. | Thermometer. | | | Barometer. | | De Luc's Hygrometer. | | Winds. | | Atmospheric Variation. | | |
|---|---|---|---|---|---|---|---|---|---|---|---|---|---|---|
| Sept. | | | a.m. | max. | min | 9a.m. | 10p.m | 9a.m. | 10p.m. | 9 a.m. | 10p.m. | 9 a. m. | 2 p.m. | 10 p. m. |
| 20 | | | 54 | 60 | 50 | 30.27 | 30.27 | 55 | 58 | WNW | NNW | fine | fine | |
| 21 | | | 56 | 62 | 55 | .29 | .26 | 60 | 63 | NW | ESE | | | |
| 22 | | | 62 | 64 | 50 | .24 | .14 | 65 | 66 | ESE | E | sleet | | |
| 23 | | | 59 | 68 | 52 | .14 | .14 | 66 | 66 | E | SSW | fine | | |
| 24 | | | 60 | 66 | 57 | .13 | .13 | 67 | 68 | SSE | SW | foggy | | |
| 25 | ● | | 62 | 70 | 56 | .17 | .12 | 68 | 68 | WSW | W | foggy | | |
| 26 | | | 61 | 70 | 54 | .07 | 29.97 | 67 | 67 | SW | SW | fine | | |
| 27 | | | 64 | 69 | 53 | 29.92 | .83 | 67 | 68 | W | SW | | | |
| 28 | | | 59 | 64 | 57 | .82 | .81 | 68 | 67 | SW | SSE | | | |
| 29 | | | 62 | 68 | 55 | .79 | .70 | 67 | 64 | SSW | SW | foggy | | |
| 30 | | | 59 | 65 | 56 | .70 | .77 | 72 | 78 | SW | SSW | cloudy | rain | |
| Oct. | | | | | | | | | | | | | | |
| 1 | | .33 | 63 | 66 | 59 | .80 | .76 | 76 | 75 | S | WSW | cloudy | fine | overcast |
| 2 | ☽ | | 61 | 64 | 55 | .66 | .78 | 75 | 74 | SSW | WSW | | | fine |
| 3 | | | 60 | 64 | 59 | .64 | .59 | 74 | 76 | SW | SW | cloudy | cloudy | overcast |
| 4 | | .25 | 62 | 65 | 55 | .53 | .46 | 75 | 80 | SW | SW | | rain | showery |
| 5 | | | 59 | 62 | 50 | .20 | .07 | 79 | 76 | S | WSW | rain | rain | fine |
| 6 | | | 56 | 56 | 52 | .30 | .35 | 74 | 73 | SW | SSW | fine | rain | rain |
| 7 | | | 55 | 61 | 48 | .18 | 28.97 | 73 | 75 | SW | SW | | cloudy | rain |
| 8 | | | 53 | 55 | 46 | .10 | 29.40 | 75 | 73 | WSW | WNW | cloudy | showery | fine |
| 9 | | 132 | 55 | 61 | 54 | .65 | .79 | 72 | 73 | NNW | WNW | fine | fine | cloudy |
| 10 | ○ | | 60 | 64 | 59 | .78 | .95 | 76 | 79 | W | WSW | cloudy | | cloudy |
| 11 | | | 63 | 65 | 60 | 30.05 | 30.05 | 80 | 77 | WSW | WSW | cloudy | cloudy | cloudy |
| 12 | | | 61 | 64 | 61 | 29.85 | 29.71 | 77 | 77 | WSW | WSW | | fine | fine |
| 13 | | | 55 | 59 | 49 | .69 | [illegible].89 | 74 | 73 | W | NW | fine | | |
| 14 | | | 52 | 61 | 47 | .92 | 30.04 | 73 | 74 | W | W | | | |
| 15 | | | 55 | 58 | 54 | 30.01 | 29.97 | 75 | 75 | W | SW | | | cloudy |
| 16 | | | 56 | 62 | 47 | 29.95 | 30.06 | 77 | 76 | WSW | NW | rain | | fine |
| 17 | ☾ | .04 | 52 | 56 | 52 | 30.13 | .11 | 75 | 76 | WNW | W | fine | | |
| 18 | | | 54 | 55 | 50 | .06 | .01 | 79 | 80 | WSW | WSW | sleet | | sleet |
| 19 | | .11 | 55 | | 42 | 30.00 | .04 | 79 | 77 | NE | NNE | rain | cloudy | fine |

The quantity of Rain fallen in September, was 85.100ths of an inch.

